# Association of brown adipose tissue activity with circulating sex hormones and fibroblast growth factor 21 in the follicular and luteal phases in young women

**DOI:** 10.1186/s40101-024-00371-6

**Published:** 2024-10-01

**Authors:** Hirokazu Taniguchi, Yuka Hashimoto, Narumi Dowaki, Shinsuke Nirengi

**Affiliations:** 1 Division of Applied Life Sciences, Graduate School of Life and Environmental Sciences, Kyoto Prefectural University, Kyoto, Japan; 2 Clinical Research Institute, Division of Preventive Medicine, National Hospital Organization Kyoto Medical Center, Kyoto, Japan; 3 Department of Physiology and Cell Biology, Dorothy M. Davis Heart and Lung Research Institute, The Ohio State University Wexner Medical Center, Columbus, OH USA

**Keywords:** Brown adipose tissue, Fibroblast growth factor 21, Thermogenesis, Estrogen, Progesterone

## Abstract

**Background:**

Thermogenesis is influenced by fluctuations in sex hormones during the menstrual cycle in premenopausal women. The thermogenic activity and mass of brown adipose tissue (BAT) are regulated by endocrine factors, including sex hormones and fibroblast growth factor 21 (FGF21). However, the relationship between human BAT and these endocrine fluctuations within individuals remains to be elucidated. This study aimed to assess variations in BAT activity between the luteal and follicular phases and identify correlations with circulating levels of sex hormones and FGF21.

**Methods:**

Healthy young women were enrolled in an observational study. Measurement of BAT activity and blood analyses were performed in both the follicular and luteal phases. BAT activity was analyzed using thermography with 2-h cold exposure. Plasma 17β-estradiol, progesterone, and FGF21 levels were determined by enzyme-linked immunosorbent assay. A comparative analysis within individuals was conducted in 13 women to compare the follicular and luteal phases. Furthermore, sensitivity analysis was carried out in 21 women during the follicular phase only.

**Results:**

Plasma 17β-estradiol and progesterone levels were significantly higher in the luteal phase, whereas plasma FGF21 level was significantly higher in the follicular phase. Comparison analysis found no significant differences in cold-induced BAT activity between the follicular and luteal phases in young women. Correlation analysis in both comparison and sensitivity analyses found that plasma 17β-estradiol and progesterone levels were not associated with BAT activity, whereas plasma FGF21 levels were significantly and positively correlated with BAT activity only in the follicular phase. In addition, plasma 17β-estradiol levels in the follicular phase were significantly and positively associated with plasma FGF21 levels in both the comparison and sensitivity analyses.

**Conclusions:**

The thermogenic activity of BAT during cold exposure was comparable between the follicular and luteal phases in young women. Higher BAT activity was associated with elevated levels of plasma FGF21 only in the follicular phase, which is related to increased plasma 17β-estradiol levels.

## Introduction

Brown adipose tissue (BAT) is mainly distributed in the supraclavicular region of adult humans [1, 2], where it increases energy expenditure by dissipating chemical energy in the form of heat [3, 4]. Acute cold exposure activates nonshivering thermogenesis in BAT, which is associated with reduced body fat [1, 5] and improved glucose metabolism [6, 7]. Interventional studies reported that BAT thermogenesis is adaptive and can be increased through the activation of the sympathetic nervous system by cold acclimation [8–10] and oral supplements [8, 11–13] in young healthy adults.

Thermogenic activity and mass of BAT are influenced by endocrine factors, as evidenced by reduced BAT thermogenesis in ovariectomized rodents [14], postmenopausal women, and premenopausal women who have suppressed ovarian function [15]. Nevertheless, evidence for endocrine effects related to the menstrual phase affecting human BAT is currently limited. A previous study reported that cold exposure for 5 min via hand immersion increased supraclavicular temperature, indicating BAT thermogenesis, in young women during the luteal phase compared with those in the follicular phase [16]. In another cross-sectional study, circulating estradiol levels in adult women were positively associated with cold-induced energy expenditure, but not with BAT activity [17]. However, these human studies did not evaluate the relationship between BAT thermogenesis and different menstrual cycle phases within individuals.

BAT thermogenesis is induced by fibroblast growth factor 21 (FGF21), which is a peptide hormone mainly secreted from the liver [18, 19]. Clinical studies reported that administration of FGF21 analogues to obese participants improved obesity, glucolipid parameters, and hepatic steatosis [20, 21]. Circulating FGF21 levels are positively correlated with thermogenic activity of BAT in young healthy men [22, 23]; however, the effects of FGF21 on BAT in women remain to be elucidated. Previous animal studies found that estrogens induce FGF21 secretion [24], thermogenesis, and browning of white adipose tissue [25, 26]. Based on these previous studies, we hypothesized that sex hormones regulate endogenous FGF21 secretion during different menstrual cycles, thereby modulating BAT thermogenesis.

This study aimed to evaluate differences in BAT activity between the luteal and follicular phases and associations with circulating levels of sex hormones and FGF21. These findings carry important implications for understanding the regulators of BAT thermogenesis in adult women.

## Materials and methods

### Participants and ethics approval

Twenty-nine young healthy women (aged 21–25 years) participated in this study. The participants were recruited at Kyoto Prefectural University through social networking services. All participants had no history of chronic diseases and pregnancy. All participants provided written informed consent, which was approved by the Ethical Committee of Kyoto Prefectural University (approval number 292). The study was conducted in accordance with the Declaration of Helsinki.

### Study design

**Fig. 1:**
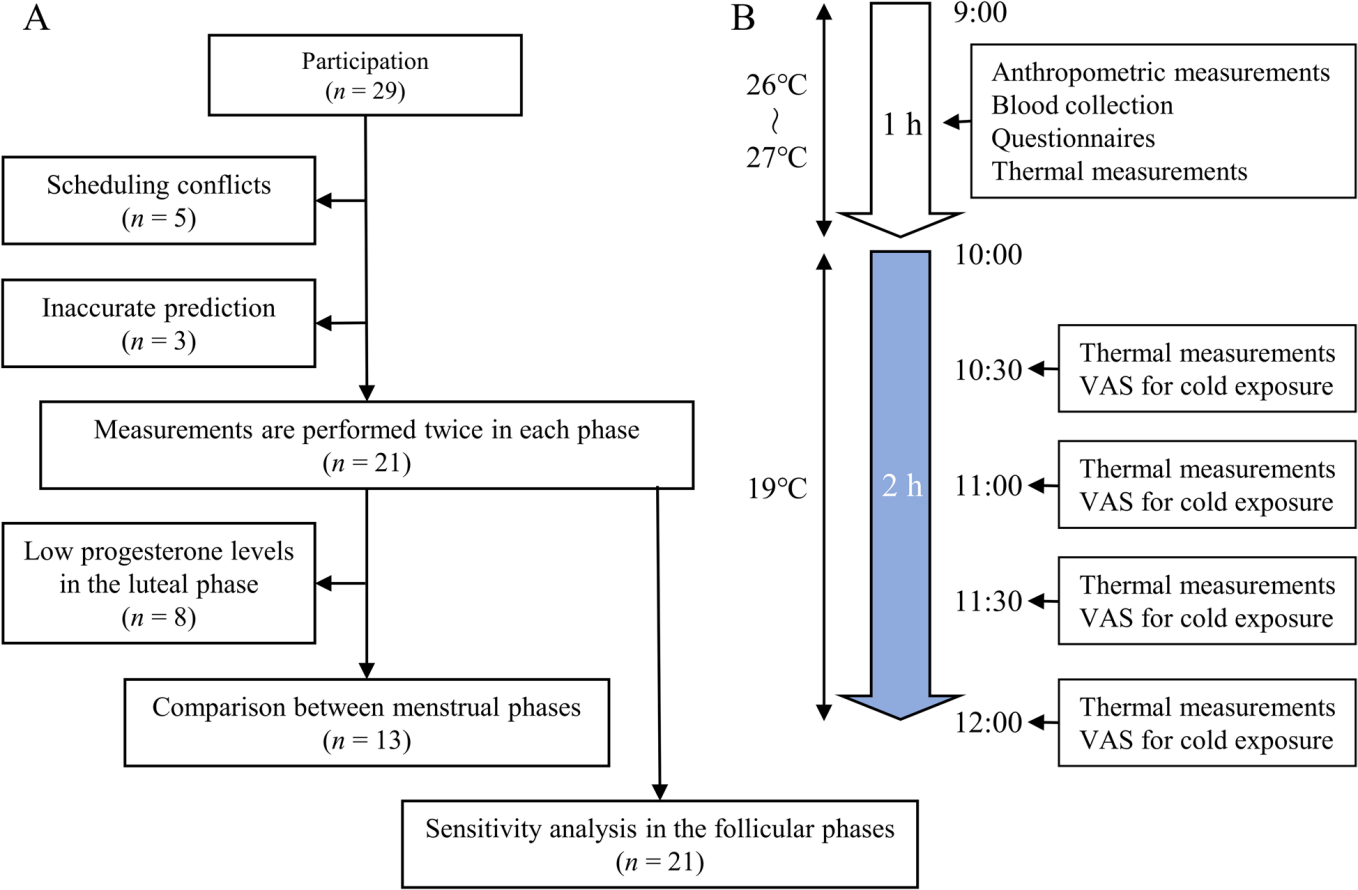
Study design. **A** Flow diagram of the participants. **B** Experimental flow of thermal measurements. VAS, visual analogue scale

We performed an observational study in the winter of 2023 (Fig. 1A). Menstrual cycle phases were predicted by the participant’s history of menstrual cycle length and their most recent menstruation. Measurements were scheduled for days 4–12 and 15–28 after the start of menstruation to occur in the predicted follicular and luteal phases, respectively. The participants were asked to measure their basal body temperature upon waking up every morning from at least 1 week before the first measurement until the final measurement. After the first measurement, eight women were excluded (five participants were unable to complete the study due to scheduling conflicts, three women due to inaccurate prediction of their menstrual cycle). Menstrual cycles were defined as increased basal body temperature and plasma progesterone levels ≥ 1.0 pg/mL in the luteal phase, leading to a further eight female participants being excluded for low plasma progesterone levels (range 0.22–0.77 pg/mL) in the luteal phase. The difference between follicular and the luteal phases was compared among the remaining 13 female participants. The association between BAT activity and plasma levels of sex hormones and FGF21 was analyzed in 21 female participants during the follicular phase as a sensitivity analysis to assess the robustness of the statistical significance [27].

### Measurement of BAT activity

BAT activity was determined by thermal imaging during the morning between 9:00 and 12:00 in December of 2023 (Fig. 1B) [23, 28, 29]. All the participants were instructed to consume their evening meal prior to 21:00 on the previous day and to skip breakfast. Body surface temperature was measured using a thermal imaging camera (DETC1000T; D-eyes, Osaka, Japan). Participants were seated at rest under thermoneutral conditions (26–27 ℃) for at least 30 min and then baseline body surface temperature was measured. An artificial climate chamber (TBRR-9A4GX; ESPEC, Osaka, Japan) set at 19 ℃ was used for cold exposure. Subjects entered the artificial climate chamber, and body surface temperature was measured at 30, 60, 90, and 120 min after mild cold exposure. Image acquisition was duplicated for each measurement. Before and during cold exposure, subjects were asked to rate their shivering, cold sensation, and discomfort using a visual analog scale [23, 28, 29].

Supraclavicular temperature was measured from a location adjacent to BAT on both the right and left sides from each image. Manubrium temperature was simultaneously measured as a control [16]. The images of body surface temperature were analyzed using a modified (D-eyes) version of the Thermal-Cam v.1.1.0.9 software (Laon People, Seoul, Korea). The average values of nine pixels in both supraclavicular and manubrium temperatures were calculated for the duplicate images. The average supraclavicular temperature minus manubrium temperature was used to estimate BAT activity. Area under the curve (AUC) of BAT activity was calculated by BAT activity before and 30, 60, 90, and 120 min after cold exposure in each menstrual phase. Maximal BAT activity was defined as the highest BAT activity during the cold exposure.

### Anthropometric characteristics and questionnaire

Body weight and body fat percentage were measured using an electronic scale (V-body HBF-359; Omron, Kyoto, Japan). Body mass index (BMI) was calculated as body weight (kg) divided by the square of height (m). Basal body temperature was measured using a basal body thermometer (MC-172L; Omron Healthcare, Kyoto, Japan). Days of regular menstrual cycle, days after menstruation, and hormonal contraceptive use were asked using a self-administered questionnaire.

### Blood analysis

Blood samples were collected under thermoneutral conditions in each menstrual phase. The collected blood samples were centrifuged at 3000 rpm for 5 min. Concentrations of plasma glucose and free fatty acids were measured using Glucose C2-test Wako and NEFA C-test Wako (FUJIFILM Wako Pure Chemical, Osaka, Japan), respectively. Serum triglycerides and total cholesterol were determined by Kyoto Biken Laboratories (Kyoto, Japan). Commercially available enzyme-linked immunosorbent assay kits were used to measure plasma levels of progesterone (RE52231; IBL International GMBH, Hamburg, Germany), 17β-estradiol (RE52041; IBL International GMBH), cortisol (RE 52061; IBL International GMBH), and FGF21 (DF2100; R&D Systems, Minneapolis, USA).

### Statistical analysis

All statistical analyses were performed using SPSS, version 29.0 (SPSS, Chicago, USA). The mean basal body temperature value for each menstrual phase was calculated based on three consecutive days of stable data. Kolmogorov–Smirnov test was performed to assess the normality of data distribution. Baseline characteristics between each menstrual phase were compared using paired Student’s *t* test for normally distributed data or Mann–Whitney *U* test for nonnormally distributed data. Two-way ANOVA analysis (time × menstrual phase) was used to determine the absolute value difference between each menstrual phase. Nonnormally distributed data were log-transformed, and relationships among variables were determined by Pearson’s correlation coefficients and partial correlation analysis adjusted for BMI. All measurements and calculated values are presented as the means ± SD (for normally distributed data) or median (interquartile range) for nonnormally distributed data. Significance was set at *p* < 0.05.

## Results

### Participant characteristics

Participant characteristics for the women in the menstrual phase comparison (*n* = 13) and sensitivity analysis (*n* = 21) are shown in Table 1. There were no significant differences in body weight, BMI, and body fat between the follicular and luteal phases. Basal body temperature was significantly higher in the luteal phase than in the follicular phase. Participants did not use oral hormonal contraception. Circulating triglycerides, free fatty acids, total cholesterol, and fasting glucose levels were not significantly different between the menstrual phases. Plasma progesterone and 17β-estradiol levels were significantly higher in the luteal phase than in the follicular phase. In the follicular phase, the participants had significantly higher levels of plasma FGF21 than those in the luteal phase. There was no significant difference in plasma cortisol levels between each menstrual cycle phase.

**Table 1:** Characteristics of the young healthy women in each menstrual cycle phase

	Comparison between menstrual phases (*n* = 13)		*p*	Sensitivity analysis (*n* = 21)
	Follicular phase	Luteal phase		Follicular phase^a^
Age (year)	22.0 (22.0–24.0)			22.0 (22.0–23.0)
Height (cm)	159.0 ± 4.6			158.4 ± 5.1
Body weight (kg)	53.3 ± 6.1	53.3 ± 6.3	0.808	51.8 ± 5.7
BMI (kg/m^2^)	21.0 ± 1.8	21.0 ± 1.9	0.841	20.6 ± 1.6
Body fat (%)	26.3 ± 2.9	26.2 ± 3.3	0.797	25.4 ± 2.9
Menstrual cycle (days)	30.0 (29.0–32.0)			30.0 (29.0–32.0)
After menstruation (days)	8.0 (6.0–14.0)	21.0 (15.0–24.0)		8.0 (7.0–12.0)
Basal body temperature (℃)	36.17 ± 0.22	36.45 ± 0.16	** < 0.001**	36.22 ± 0.25
Triglycerides (mg/dL)	63.8 ± 24.2	54.8 ± 21.0	0.110	67.1 ± 27.8
Free fatty acids (mEq/L)	0.28 ± 0.09	0.29 ± 0.12	0.860	0.29 ± 0.10
Total cholesterol (mg/dL)	176.0 (169.0–191.0)	168.0 (165.0–175.0)	0.071	170.0 (167.0–191.0)
Fasting glucose (mg/dL)	85.6 (80.7–86.7)	82.5 (78.4–88.6)	0.807	86.2 (80.7–88.5)
Progesterone (ng/mL)	0.66 (0.38–0.76)	7.48 (2.56–12.42)	**0.001**	0.53 (0.38–0.73)
17β-estradiol (pg/mL)	95.6 (89.0–122.8)	165.1 (120.4–182.2)	**0.016**	93.0 (72.7–104.7)
Cortisol (µg/mL)	138.8 (118.5–180.3)	124.1 (97.6–175.5)	0.552	147.0 (104.7–180.3)
FGF21 (pg/mL)	156.3 (110.1–321.7)	98.5 (77.2–179.4)	**0.023**	156.3 (105.9–308.8)

Data are the mean ± SD or median (interquartile range) values. Boldface indicates significance

*BMI* body mass index, *FGF21* fibroblast growth factor 21

^a^Three women who used oral contraceptive pills were included

In the sensitivity analysis, 21 women, including eight excluded participants, were analyzed. They had slightly lower anthropometric characteristics including BMI and body fat. Three of the eight excluded women used oral hormonal contraception to manage their menstrual cycles or prevent pregnancy. In the follicular phase, plasma levels of sex hormones and FGF21 were comparable between the comparison analysis (*n* = 13) and sensitivity analysis (*n* = 21).

### Cold-induced BAT activity between the follicular and luteal phases

**Fig. 2:**
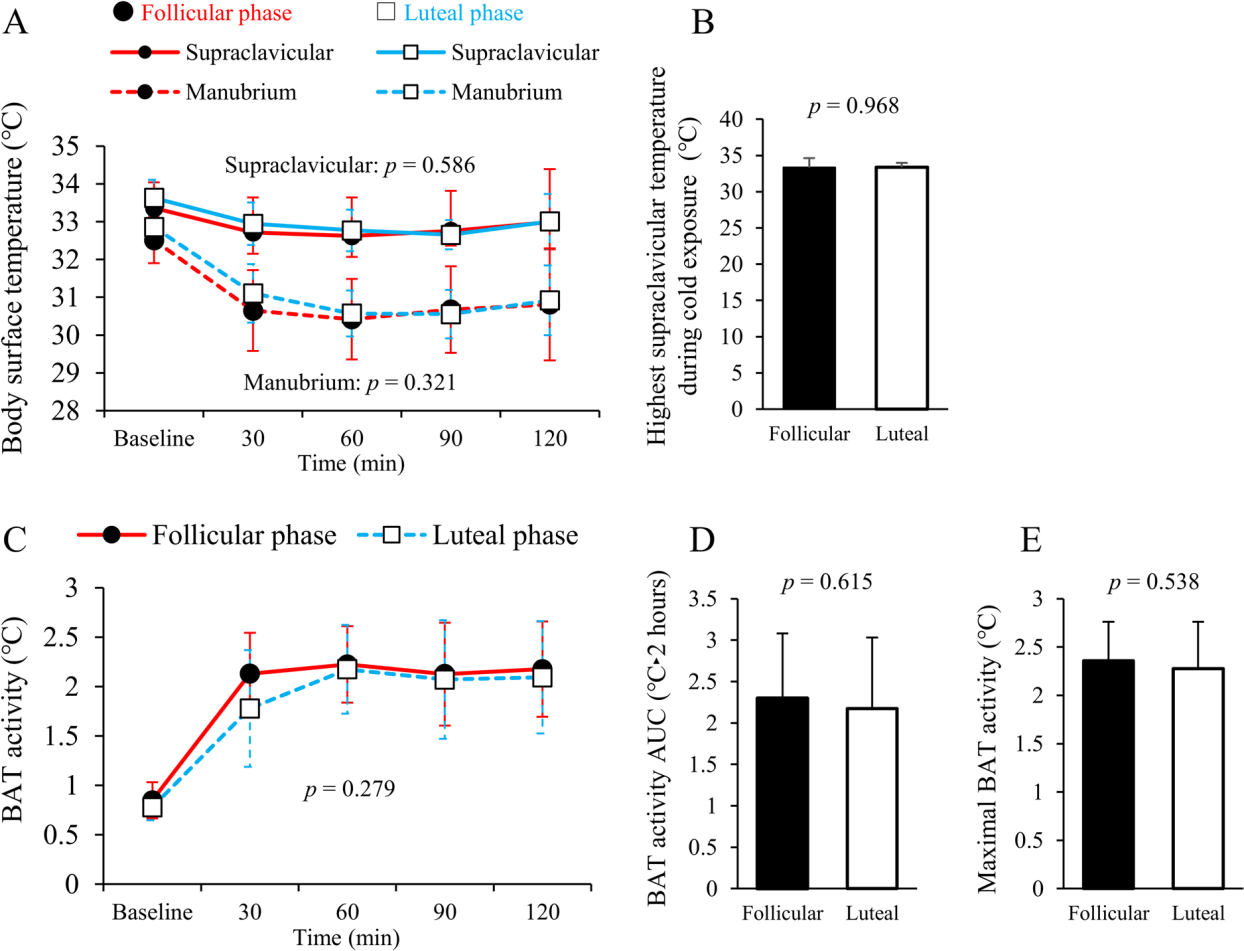
BAT activity in the follicular and luteal phases. **A** Changes in supraclavicular and manubrium temperatures before and during cold exposure. **B** Highest supraclavicular temperature during cold exposure. **C** Changes in BAT activity before and during cold exposure. **D** BAT activity AUC. **E** Maximal BAT activity. Two-way ANOVA analysis and paired Student’s t test were performed. AUC, area under the curve; BAT, brown adipose tissue

Figure 2A shows supraclavicular and manubrium temperatures before and during cold exposure of 13 female participants. Mann–Whitney *U* test at each time point found that shivering response, cold sensation, and discomfort were not significantly different between each menstrual phase (*p* > 0.05). Two-way ANOVA found no significant difference in the supraclavicular and manubrium temperatures between each menstrual cycle phase. The highest supraclavicular temperatures were not significantly different between the phases during cold exposure (Fig. 2B).

The data regarding BAT activity before and during cold exposure are shown in Fig. 2C. Although higher average values of BAT activity were observed 30 min after cold exposure in the follicular phase (2.13 ± 0.42 ℃) compared with the luteal phase (1.78 ± 0.59 ℃), two-way ANOVA found that BAT activity was not significantly different between the phases during cold exposure. The Kolmogorov–Smirnov test showed normal distributions of BAT activity AUC and maximal BAT activity. There was no significant difference in the BAT activity AUC (Fig. 2D) and maximal BAT activity (Fig. 2E) between each menstrual cycle phase.

### BAT activity correlation with plasma FGF21 levels in the follicular phase

**Fig. 3:**
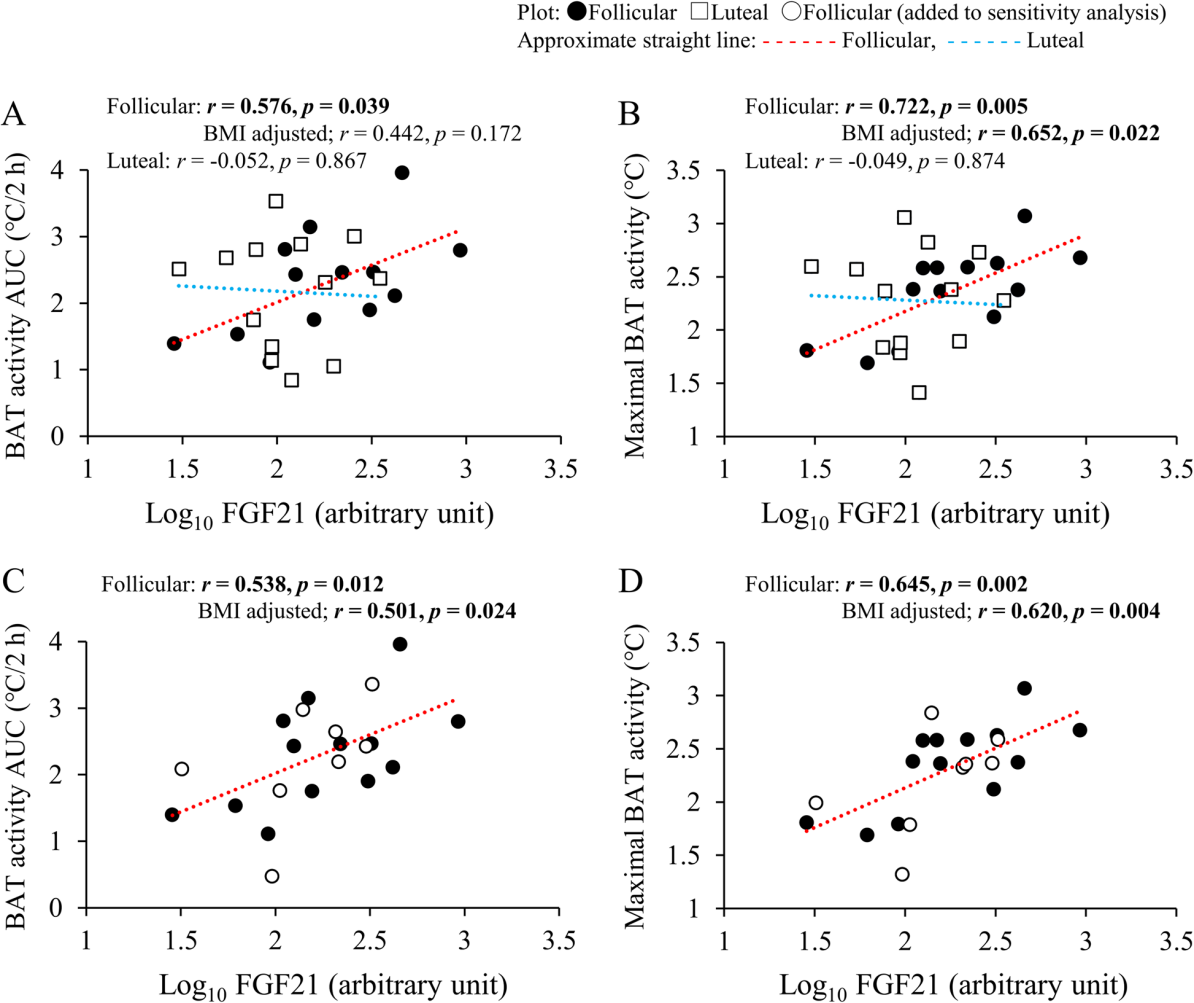
Correlation analyses with plasma FGF21 levels. **A** BAT activity AUC in each menstrual phase (*n* = 13). **B** Maximal BAT activity in each menstrual phase (*n* = 13). **C** BAT activity AUC in the follicular phase (*n* = 21). **D** Maximal BAT activity in the follicular phase (*n* = 21). Boldface indicates significance. AUC, area under the curve; BAT, brown adipose tissue; BMI, body mass index; FGF21, fibroblast growth factor 21

**Fig. 4:**
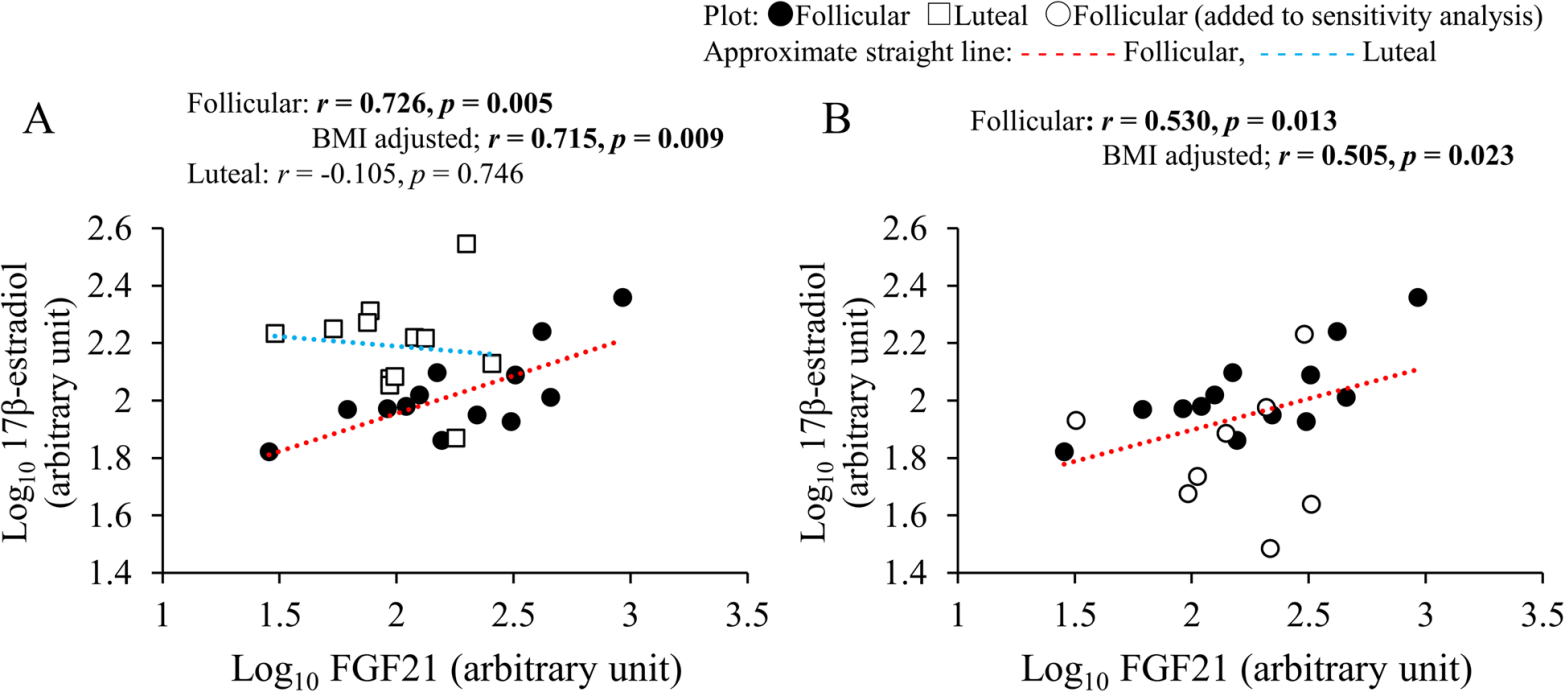
Correlation analyses between plasma FGF21 and 17β-estradiol levels. **A** Correlation in each menstrual phase (*n* = 13). **B** Correlation in the follicular phase (*n* = 21). Boldface indicates significance. AUC, area under the curve; BAT, brown adipose tissue; BMI, body mass index; FGF21, fibroblast growth factor 21

The results of Pearson’s correlation analysis in each menstrual phase (*n* = 13) are shown in Table 2. In the follicular phase, both BAT activity AUC and maximal BAT activity were significantly and positively correlated with BMI and plasma FGF21 levels (Fig. 3A and B). Partial correlation analysis found that there was significant positive correlation between plasma FGF21 levels and maximal BAT activity after adjustment for BMI (Fig. 3B). Body fat percentage was positively correlated with maximal BAT activity, whereas plasma cortisol level was negatively correlated with BAT activity AUC in the follicular phase. Correlation analysis among plasma hormones showed that plasma 17β-estradiol level was significantly and positively correlated with plasma FGF21 level in the follicular phase even after adjustment for BMI (Fig. 4A).

**Table 2:** Correlation of BAT activity with other variables for healthy young women in each menstrual cycle phase (*n* = 13)

	Follicular phase				Luteal phase			
	BAT activity AUC		Maximal BAT activity		BAT activity AUC		Maximal BAT activity	
	*r*	*p*	*r*	*p*	*r*	*p*	*r*	*p*
BMI (kg/m^2^)	**0.875**	** < 0.001**	**0.752**	**0.003**	0.138	0.654	0.135	0.659
Body fat (%)	0.467	0.108	0.500	0.082	0.091	0.767	0.065	0.833
Basal body temperature (℃)	0.221	0.469	0.164	0.593	0.492	0.088	0.474	0.101
Triglycerides (mg/dL)	− 0.070	0.821	− 0.218	0.473	− 0.085	0.784	0.031	0.921
Free fatty acids (mEq/L)	− 0.027	0.930	− 0.104	0.736	0.086	0.781	0.162	0.598
Log_10_ total cholesterol	0.020	0.949	− 0.178	0.562	− 0.167	0.587	− 0.263	0.386
Log_10_ fasting glucose	0.445	0.128	0.225	0.459	0.412	0.162	0.181	0.553
Log_10_ progesterone	− 0.428	0.144	− 0.446	0.127	0.305	0.311	0.275	0.363
Log_10_ 17β-estradiol^a^	0.405	0.170	0.445	0.128	− 0.210	0.513	− 0.191	0.552
Log_10_ cortisol	− 0.496	0.085	− 0.459	0.114	− 0.266	0.380	− 0.288	0.340
Log_10_ FGF21	**0.576**	**0.039**	**0.722**	**0.005**	− 0.052	0.867	− 0.049	0.874

Data are the Pearson’s correlation coefficients. Boldface indicates significance

The total cholesterol, fasting glucose, progesterone, 17β-estradiol, cortisol, and FGF21 data were log transformed prior to analysis. These variables were presented in arbitrary units

*AUC* area under the curve, *BAT* brown adipose tissue, *BMI* body mass index, *FGF21* fibroblast growth factor 21

^a^A participant with an abnormal 17β-estradiol level was excluded from the correlation (*n* = 12)

One participant with an elevated plasma 17β-estradiol level (1272 pg/mL) was excluded from the luteal phase correlation analysis. In the luteal phase, basal body temperature was positively associated with BAT activity AUC (Table 2). Indices of BAT activity demonstrated no significant correlation with other variables, including FGF21 (Fig. 3A and B). There was no significant correlation between plasma FGF21 and 17β-estradiol levels in the luteal phase (Fig. 4A).

### Sensitivity analysis of robustness between BAT activity and FGF21 in the follicular phase

To evaluate the statistical robustness of the findings, we conducted correlation analyses on a total of 21 women in the follicular phase. The data included the 13 women who were included in the comparison analysis and eight female participants with lower progesterone levels in the luteal phase (Fig. 1A and Table 1).

Table 3 shows the results of the sensitivity analysis. In the follicular phase, BMI tended to be positively correlated with BAT activity AUC, but not with maximal BAT activity. Only plasma FGF21 levels were significantly and positively correlated with BAT activity AUC (Fig. 3C) and maximal BAT activity (Fig. 3D), even after BMI adjustment. There was a significant positive correlation between plasma FGF21 and 17β-estradiol levels in both the single and partial correlation analyses (Fig. 4B).

**Table 3:** Sensitivity analysis: correlation of BAT activity with other variables for healthy young women in follicular phase (*n* = 21)

	Follicular phase							
	Simple correlation				Partial correlation adjusted for BMI			
	BAT activity AUC		Maximal BAT activity		BAT activity AUC		Maximal BAT activity	
	*r*	*p*	*r*	*p*	*r*	*p*	*r*	*p*
BMI (kg/m^2^)	0.405	0.068	0.350	0.120				
Body fat (%)	0.065	0.779	0.109	0.639	− 0.274	0.242	− 0.158	0.506
Basal body temperature (℃)	0.093	0.689	0.008	0.973	0.001	0.997	− 0.078	0.742
Triglycerides (mg/dL)	− 0.040	0.865	− 0.135	0.561	− 0.089	0.709	− 0.183	0.440
Free fatty acids (mEq/L)	0.108	0.640	0.138	0.552	0.058	0.807	0.097	0.685
Log_10_ total cholesterol	0.225	0.326	0.041	0.860	0.291	0.213	0.080	0.736
Log_10_ fasting glucose	0.305	0.179	0.273	0.232	0.162	0.494	0.154	0.518
Log_10_ progesterone	− 0.284	0.212	− 0.219	0.341	− 0.218	0.356	− 0.154	0.516
Log_10_ 17β-Estradiol	0.273	0.231	0.353	0.117	0.202	0.392	0.299	0.201
Log_10_ cortisol	− 0.148	0.521	− 0.137	0.553	0.022	0.928	0.007	0.975
Log_10_ FGF21	**0.538**	**0.012**	**0.645**	**0.002**	**0.501**	**0.024**	**0.620**	**0.004**

Data are Pearson’s correlation coefficients and partial correlation coefficients. Boldface indicates statistical significance

Total cholesterol, fasting glucose, progesterone, 17β-estradiol, cortisol, and FGF21 data were log transformed prior to analysis. These variables were presented in arbitrary units

*AUC* area under the curve, *BAT* brown adipose tissue, *BMI* body mass index, *FGF21* fibroblast growth factor 21

## Discussion

The indices of BAT activity, including AUC_2h_ and maximal values, were not significantly different between the follicular and luteal phases among young women. These findings suggest that BAT thermogenesis is maintained during different menstrual cycles. Correlation analysis revealed no association between BAT activity and sex hormones, but a positive correlation between plasma FGF21 levels and BAT activity only in the follicular phase. Thus, circulating FGF21 plays a regulatory role in BAT activity during the follicular phase for premenopausal women. In addition, plasma 17β-estradiol levels in the follicular phase were positively associated with plasma FGF21 levels. This suggests that 17β-estradiol induces FGF21 secretion, which supports the findings of a previous animal study [24].

Healthy premenopausal women have higher core body temperatures and energy expenditure during the luteal phase than the follicular phase. Previous studies reported that thermogenic changes are regulated antagonistically by sex hormones [30, 31]. Early studies of women reported that core body temperature was increased by progesterone administration [30], whereas it was decreased by estrogen administration [32, 33]. A previous study of endocrine patterns of sex hormones reported that estrogens and progesterone increase at the start of the follicular and luteal phases, respectively [34]. Moreover, progesterone level is lower in the follicular phase, whereas estrogen secretion is maintained in the luteal phase [34]. The observed differences in plasma progesterone and basal body temperature between the phases in the present study were consistent with typical menstrual cycles. Although it is generally accepted that the highest levels of circulating estrogen are observed in the late follicular phase [34], we found that plasma 17β-estradiol levels were higher in the luteal phase; therefore, our dataset may have included some women in the early stage of the follicular phase. Despite the difference in body temperature and sex hormones, BAT activity was not significantly different between the follicular and luteal phases. Thus, our findings suggest that female BAT activity was stable within individuals throughout their menstrual cycles.

The effects of sex hormones on BAT thermogenesis are controversial [31]. Some studies reported that BAT, which is located near the body surface, is inversely regulated by sex hormones compared with core body temperature. This is supported by studies on ovariectomized rodents that reported that estrogen increased BAT thermogenesis [35], whereas progesterone decreased the expression of BAT markers [36]. However, our correlation analysis found no association between BAT activity and sex hormones in young women, and thus the effects of sex hormones on BAT thermogenesis may only be observed in ovariectomized and/or menopausal conditions. In the present study, BAT activity was positively correlated with plasma FGF21 levels, which supports studies of young men [22, 23]; however, this was only observed in the follicular phase in young women. Moreover, we found a positive correlation between plasma FGF21 and 17β-estradiol levels. These findings suggest that sex hormones modulate BAT thermogenesis through increased FGF21 secretion. Thus, estrogens stimulate FGF21 secretion, which in turn induces BAT thermogenesis instead of decreasing the core body temperature during the follicular phase. Because BAT activity induced by estrogens/FGF21 may plateau during the follicular phase, such a correlation may not be observed in the luteal phase between FGF21 and BAT activity, or between FGF21 and 17β-estradiol.

A previous study reported that ovariectomized rats exhibited a decrease in hepatic and plasma concentrations of FGF21, which was accompanied by visceral/hepatic fat accumulation [37]. In another study, treatment with 17β-estradiol increased energy expenditure and prevented fat accumulation in ovariectomized female mice; however, these beneficial effects were not observed in FGF21-deficient mice [26]. This suggests that sex hormones play an important role in FGF21-induced lipolysis in females, and this may be linked to a higher risk of visceral adiposity in postmenopausal women [38]. It was reported that obesity reduces sensitivity to FGF21, resulting in increased levels of circulating FGF21 at rest, which is considered a state of FGF21 resistance [39]. The discrepancy between the function of FGF21 and its circulating level has been observed in previous human studies, which reported a positive correlation between serum FGF21 levels and BMI, as well as visceral fat area [40, 41]. As 17β-estradiol treatment was associated with lower serum FGF21 concentrations in ovariectomized women [26], FGF21 resistance may be improved by hormone therapy in women with ovarian failure. A previous study reported that FGF21-resistant state was improved by endurance exercise in older individuals regardless of body weight change [42]. Thus, a combination of hormone therapy and exercise may prevent metabolic disorders in such women.

A previous cross-sectional study reported a negative correlation between supraclavicular temperature after 5-min cold exposure via hand immersion and serum cortisol levels [16]. It also found that cold-induced supraclavicular temperatures were higher in women during the luteal phase than in those in the follicular phase. However, the present study did not find a significant association between BAT activity and either cortisol levels or menstrual phases. The cortisol levels in our study were higher during the follicular phase; however, the difference was not significant. A previous meta-analysis reported significantly higher cortisol levels in the follicular phase compared to the luteal phase [43]. Thus, the negative correlation between supraclavicular temperature and cortisol levels may be attributed to the inclusion of female participants who exhibited higher supraclavicular temperatures and lower cortisol levels during the luteal phase.

Positron emission tomography-computerized tomography studies reported that BAT activity was generally associated with lower BMI [44, 45]. On the other hand, BAT activity in the follicular phase was positively correlated with BMI in the present comparison analysis (*n* = 13) but not significant in the sensitivity analysis (*n* = 21). These findings may be attributed to the small sample size in this study. Previous research has reported a correlation between BAT activity and BMI in individuals of both sexes and with diverse characteristics, including variations in age and BMI [44, 45]. Thus, an additional potential explanation is the narrow range of characteristics observed in the young healthy women included in the present study. In addition, these previous studies did not evaluate whether the association between BAT activity and BMI is affected by menstrual phases. As FGF21 levels are positively associated with both BMI and BAT activity, the observed indirect correlation between BMI and BAT activity in the follicular phase may be attributed to the influence of FGF21. The determinants of BAT activity in young women remain to be fully elucidated; thus, further studies on a larger scale are needed to validate the results of this study. Conversely, a significant correlation between plasma FGF21 levels and BAT activity was observed during the follicular phase in the sensitivity analysis. As the result did not change after adjustment for BMI in both the comparison and sensitivity analyses, this suggests that the association between FGF21 and BAT activity was robust in young healthy women.

The present study has several limitations. First, the sample size was relatively small. Second, BAT activity was not assessed using positron emission tomography-computerized tomography, which is considered the gold standard for evaluating BAT activity [3, 46]. A previous study found no significant difference between menstrual phases in terms of oxygen uptake and respiratory quotient during cold water immersion in young women [47]. However, evaluating additional metabolic variables, such as respiratory parameters, may provide further insight into whether the menstrual cycle affects both BAT activity and overall energy utilization. Third, our findings may not be generalizable to other age categories and patients with metabolic diseases. Fourth, the measurements were taken at a single time point during each menstrual cycle. Interventional studies are required to evaluate the causal relationship of estrogens with FGF21 secretion and BAT activity.

## Conclusions

There was no difference in BAT thermogenesis between the follicular and luteal phases in young Japanese women. In the follicular phase, BAT activity is positively correlated with plasma FGF21 levels, which are associated with increased plasma 17β-estradiol levels. The findings of this study indicate a need for further studies into the endocrine regulation of BAT thermogenesis in female subjects.

## Data Availability

The datasets used and/or analyzed during the present study are available from the corresponding author on reasonable request.

## Abbreviations

AUC: Area under the curve
BMI: Body mass index
BAT: Brown adipose tissue
FGF21: Fibroblast growth factor 21
